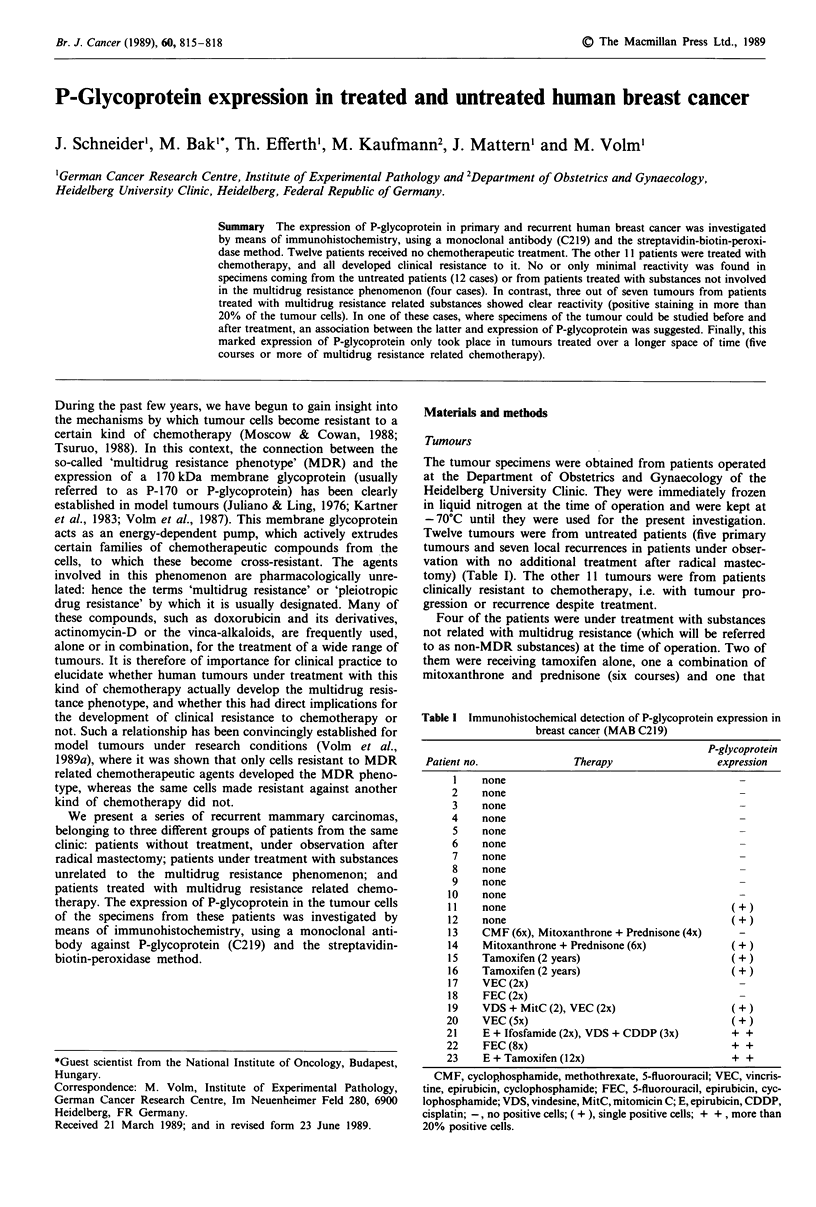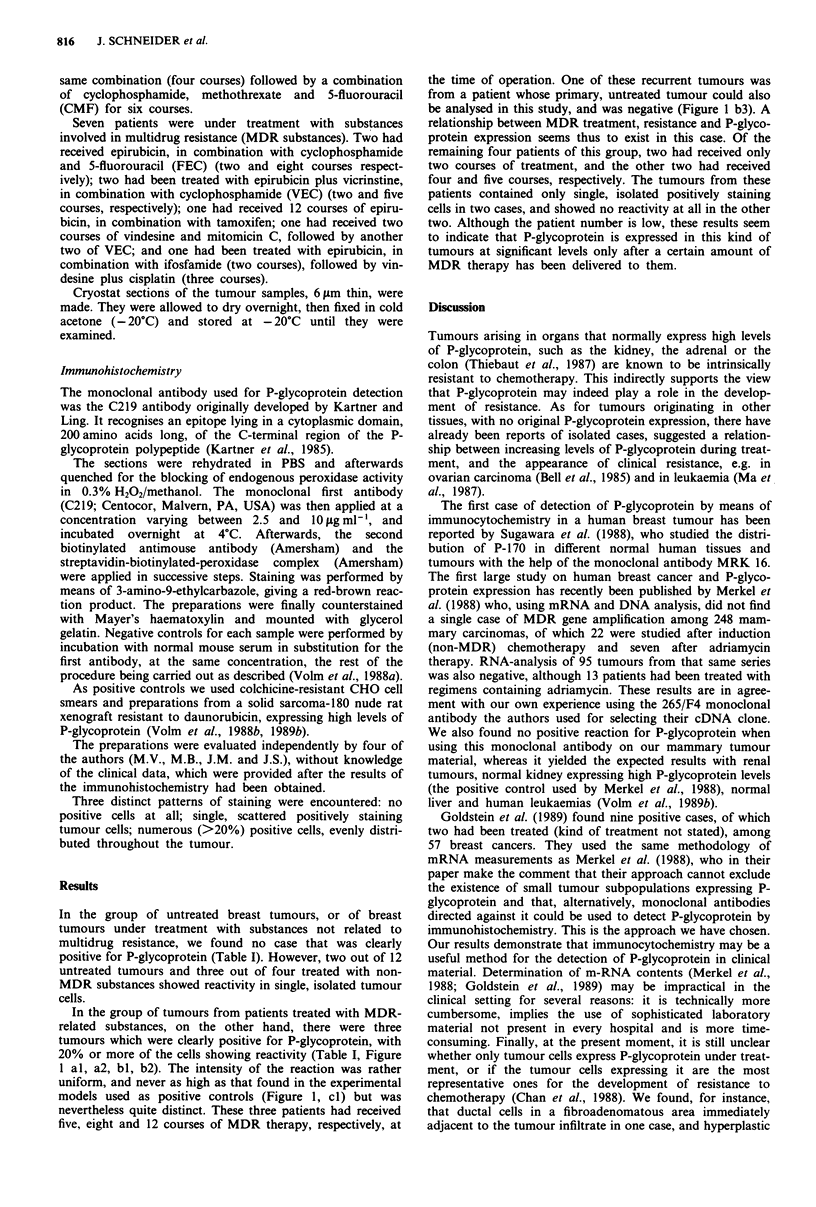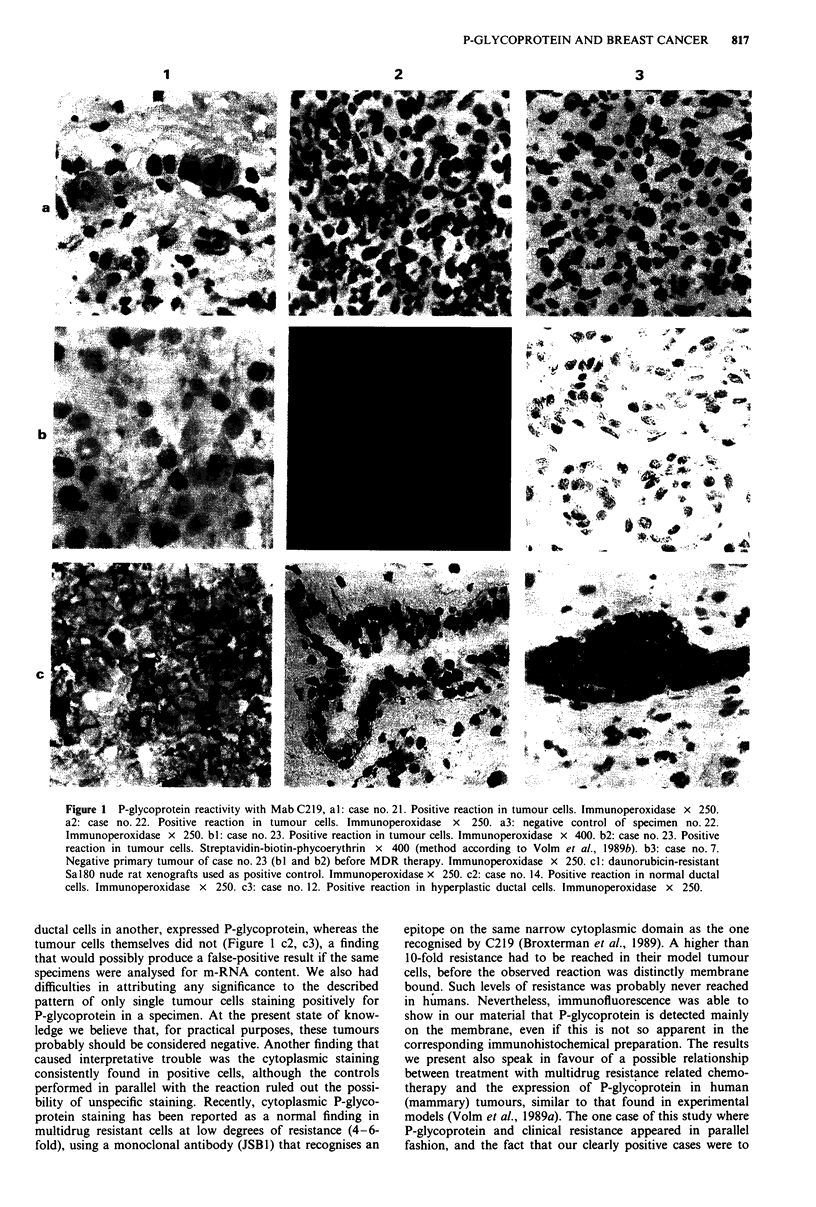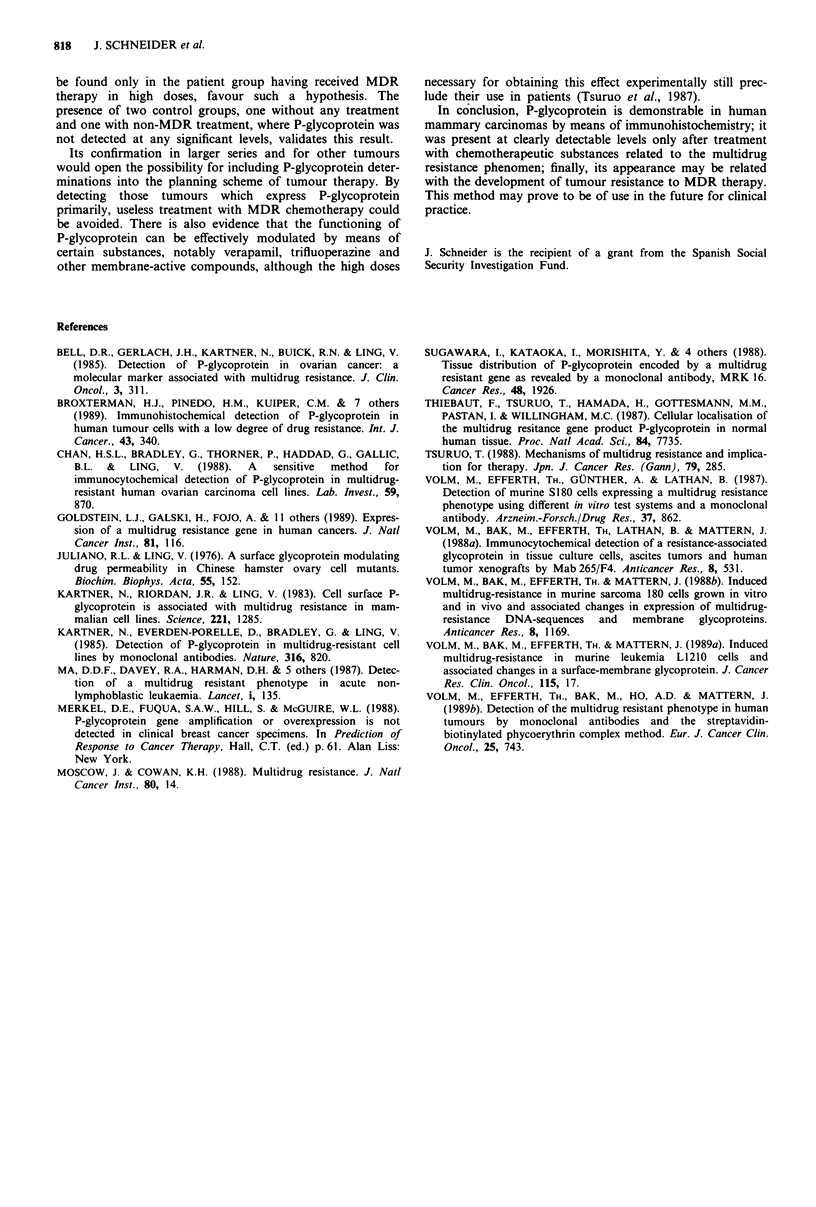# P-glycoprotein expression in treated and untreated human breast cancer.

**DOI:** 10.1038/bjc.1989.372

**Published:** 1989-12

**Authors:** J. Schneider, M. Bak, T. Efferth, M. Kaufmann, J. Mattern, M. Volm

**Affiliations:** German Cancer Research Centre, Institute of Experimental Pathology, Heidelberg.

## Abstract

**Images:**


Br. J. Cancer (1989), 60, 815-818 © The Macmillan Press Ltd., 1989

### P-Glycoprotein expression in treated and untreated human breast cancer

J. Schneider[1], M. Bak[1*], Th. Efferth[1], M. Kaufmann[2], J. Mattern[1] and M. Volm[1]

[1]German Cancer Research Centre, Institute of Experimental Pathology and [2]Department of Obstetrics and Gynaecology, Heidelberg University Clinic, Heidelberg, Federal Republic of Germany.

**Summary** The expression of P-glycoprotein in primary and recurrent human breast cancer was investigated by means of immunohistochemistry, using a monoclonal antibody (C219) and the streptavidin-biotin-peroxidase method. Twelve patients received no chemotherapeutic treatment. The other 11 patients were treated with chemotherapy, and all developed clinical resistance to it. No or only minimal reactivity was found in specimens coming from the untreated patients (12 cases) or from patients treated with substances not involved in the multidrug resistance phenomenon (four cases). In contrast, three out of seven tumours from patients treated with multidrug resistance related substances showed clear reactivity (positive staining in more than 20% of the tumour cells). In one of these cases, where specimens of the tumour could be studied before and after treatment, an association between the latter and expression of P-glycoprotein was suggested. Finally, this marked expression of P-glycoprotein only took place in tumours treated over a longer space of time (five courses or more of multidrug resistance related chemotherapy).

During the past few years, we have begun to gain insight into the mechanisms by which tumour cells become resistant to a certain kind of chemotherapy (Moscow & Cowan, 1988; Tsuruo, 1988). In this context, the connection between the so-called 'multidrug resistance phenotype' (MDR) and the expression of a 170 kDa membrane glycoprotein (usually referred to as P-170 or P-glycoprotein) has been clearly established in model tumours (Juliano & Ling, 1976; Kartner *et al.*, 1983; Volm *et al.*, 1987). This membrane glycoprotein acts as an energy-dependent pump, which actively extrudes certain families of chemotherapeutic compounds from the cells, to which these become cross-resistant. The agents involved in this phenomenon are pharmacologically unrelated: hence the terms 'multidrug resistance' or 'pleiotropic drug resistance' by which it is usually designated. Many of these compounds, such as doxorubicin and its derivatives, actinomycin-D or the vinca-alkaloids, are frequently used, alone or in combination, for the treatment of a wide range of tumours. It is therefore of importance for clinical practice to elucidate whether human tumours under treatment with this kind of chemotherapy actually develop the multidrug resistance phenotype, and whether this had direct implications for the development of clinical resistance to chemotherapy or not. Such a relationship has been convincingly established for model tumours under research conditions (Volm *et al.*, 1989*a*), where it was shown that only cells resistant to MDR related chemotherapeutic agents developed the MDR phenotype, whereas the same cells made resistant against another kind of chemotherapy did not.

We present a series of recurrent mammary carcinomas, belonging to three different groups of patients from the same clinic: patients without treatment, under observation after radical mastectomy; patients under treatment with substances unrelated to the multidrug resistance phenomenon; and patients treated with multidrug resistance related chemotherapy. The expression of P-glycoprotein in the tumour cells of the specimens from these patients was investigated by means of immunohistochemistry, using a monoclonal antibody against P-glycoprotein (C219) and the streptavidin-biotin-peroxidase method.

*Guest scientist from the National Institute of Oncology, Budapest, Hungary.

Correspondence: M. Volm, Institute of Experimental Pathology, German Cancer Research Centre, Im Neuenheimer Feld 280, 6900 Heidelberg, FR Germany.

Received 21 March 1989; and in revised form 23 June 1989.

### Materials and methods

### *Tumours*

The tumour specimens were obtained from patients operated at the Department of Obstetrics and Gynaecology of the Heidelberg University Clinic. They were immediately frozen in liquid nitrogen at the time of operation and were kept at −70°C until they were used for the present investigation. Twelve tumours were from untreated patients (five primary tumours and seven local recurrences in patients under observation with no additional treatment after radical mastectomy) (Table I). The other 11 tumours were from patients clinically resistant to chemotherapy, i.e. with tumour progression or recurrence despite treatment.

Four of the patients were under treatment with substances not related with multidrug resistance (which will be referred to as non-MDR substances) at the time of operation. Two of them were receiving tamoxifen alone, one a combination of mitoxanthrone and prednisone (six courses) and one that

**Table I** Immunohistochemical detection of P-glycoprotein expression in breast cancer (MAB C219)

| *Patient no.* | *Therapy* | *P-glycoprotein expression* |
|---|---|---|
| 1 | none | – |
| 2 | none | – |
| 3 | none | – |
| 4 | none | – |
| 5 | none | – |
| 6 | none | – |
| 7 | none | – |
| 8 | none | – |
| 9 | none | – |
| 10 | none | – |
| 11 | none | (+) |
| 12 | none | (+) |
| 13 | CMF (6x), Mitoxanthrone + Prednisone (4x) | – |
| 14 | Mitoxanthrone + Prednisone (6x) | (+) |
| 15 | Tamoxifen (2 years) | (+) |
| 16 | Tamoxifen (2 years) | (+) |
| 17 | VEC (2x) | – |
| 18 | FEC (2x) | – |
| 19 | VDS + MitC (2), VEC (2x) | (+) |
| 20 | VEC (5x) | (+) |
| 21 | E + Ifosfamide (2x), VDS + CDDP (3x) | + + |
| 22 | FEC (8x) | + + |
| 23 | E + Tamoxifen (12x) | + + |

CMF, cyclophosphamide, methothrexate, 5-fluorouracil; VEC, vincristine, epirubicin, cyclophosphamide; FEC, 5-fluorouracil, epirubicin, cyclophosphamide; VDS, vindesine, MitC, mitomicin C; E, epirubicin, CDDP, cisplatin; –, no positive cells; ( + ), single positive cells; + +, more than 20% positive cells.

same combination (four courses) followed by a combination of cyclophosphamide, methothrexate and 5-fluorouracil (CMF) for six courses.

Seven patients were under treatment with substances involved in multidrug resistance (MDR substances). Two had received epirubicin, in combination with cyclophosphamide and 5-fluorouracil (FEC) (two and eight courses respectively); two had been treated with epirubicin plus vicrinstine, in combination with cyclophosphamide (VEC) (two and five courses, respectively); one had received 12 courses of epirubicin, in combination with tamoxifen; one had received two courses of vindesine and mitomicin C, followed by another two of VEÇ; and one had been treated with epirubicin, in combination with ifosfamide (two courses), followed by vindesine plus cisplatin (three courses).

Cryostat sections of the tumour samples, 6 μm thin, were made. They were allowed to dry overnight, then fixed in cold acetone (−20°C) and stored at −20°C until they were examined.

### Immunohistochemistry

The monoclonal antibody used for P-glycoprotein detection was the C219 antibody originally developed by Kartner and Ling. It recognises an epitope lying in a cytoplasmic domain, 200 amino acids long, of the C-terminal region of the P-glycoprotein polypeptide (Kartner et al., 1985).

The sections were rehydrated in PBS and afterwards quenched for the blocking of endogenous peroxidase activity in 0.3% $H_2O_2$/methanol. The monoclonal first antibody (C219; Centocor, Malvern, PA, USA) was then applied at a concentration varying between 2.5 and 10 $\mu g\,ml^{-1}$, and incubated overnight at 4°C. Afterwards, the second biotinylated antimouse antibody (Amersham) and the streptavidin-biotinylated-peroxidase complex (Amersham) were applied in successive steps. Staining was performed by means of 3-amino-9-ethylcarbazole, giving a red-brown reaction product. The preparations were finally counterstained with Mayer's haematoxylin and mounted with glycerol gelatin. Negative controls for each sample were performed by incubation with normal mouse serum in substitution for the first antibody, at the same concentration, the rest of the procedure being carried out as described (Volm et al., 1988a).

As positive controls we used colchicine-resistant CHO cell smears and preparations from a solid sarcoma-180 nude rat xenograft resistant to daunorubicin, expressing high levels of P-glycoprotein (Volm et al., 1988b, 1989b).

The preparations were evaluated independently by four of the authors (M.V., M.B., J.M. and J.S.), without knowledge of the clinical data, which were provided after the results of the immunohistochemistry had been obtained.

Three distinct patterns of staining were encountered: no positive cells at all; single, scattered positively staining tumour cells; numerous (>20%) positive cells, evenly distributed throughout the tumour.

### Results

In the group of untreated breast tumours, or of breast tumours under treatment with substances not related to multidrug resistance, we found no case that was clearly positive for P-glycoprotein (Table I). However, two out of 12 untreated tumours and three out of four treated with non-MDR substances showed reactivity in single, isolated tumour cells.

In the group of tumours from patients treated with MDR-related substances, on the other hand, there were three tumours which were clearly positive for P-glycoprotein, with 20% or more of the cells showing reactivity (Table I, Figure 1 a1, a2, b1, b2). The intensity of the reaction was rather uniform, and never as high as that found in the experimental models used as positive controls (Figure 1, c1) but was nevertheless quite distinct. These three patients had received five, eight and 12 courses of MDR therapy, respectively, at the time of operation. One of these recurrent tumours was from a patient whose primary, untreated tumour could also be analysed in this study, and was negative (Figure 1 b3). A relationship between MDR treatment, resistance and P-glycoprotein expression seems thus to exist in this case. Of the remaining four patients of this group, two had received only two courses of treatment, and the other two had received four and five courses, respectively. The tumours from these patients contained only single, isolated positively staining cells in two cases, and showed no reactivity at all in the other two. Although the patient number is low, these results seem to indicate that P-glycoprotein is expressed in this kind of tumours at significant levels only after a certain amount of MDR therapy has been delivered to them.

### Discussion

Tumours arising in organs that normally express high levels of P-glycoprotein, such as the kidney, the adrenal or the colon (Thiebaut et al., 1987) are known to be intrinsically resistant to chemotherapy. This indirectly supports the view that P-glycoprotein may indeed play a role in the development of resistance. As for tumours originating in other tissues, with no original P-glycoprotein expression, there have already been reports of isolated cases, suggested a relationship between increasing levels of P-glycoprotein during treatment, and the appearance of clinical resistance, e.g. in ovarian carcinoma (Bell et al., 1985) and in leukaemia (Ma et al., 1987).

The first case of detection of P-glycoprotein by means of immunocytochemistry in a human breast tumour has been reported by Sugawara et al. (1988), who studied the distribution of P-170 in different normal human tissues and tumours with the help of the monoclonal antibody MRK 16. The first large study on human breast cancer and P-glycoprotein expression has recently been published by Merkel et al. (1988) who, using mRNA and DNA analysis, did not find a single case of MDR gene amplification among 248 mammary carcinomas, of which 22 were studied after induction (non-MDR) chemotherapy and seven after adriamycin therapy. RNA-analysis of 95 tumours from that same series was also negative, although 13 patients had been treated with regimens containing adriamycin. These results are in agreement with our own experience using the 265/F4 monoclonal antibody the authors used for selecting their cDNA clone. We also found no positive reaction for P-glycoprotein when using this monoclonal antibody on our mammary tumour material, whereas it yielded the expected results with renal tumours, normal kidney expressing high P-glycoprotein levels (the positive control used by Merkel et al., 1988), normal liver and human leukaemias (Volm et al., 1989b).

Goldstein et al. (1989) found nine positive cases, of which two had been treated (kind of treatment not stated), among 57 breast cancers. They used the same methodology of mRNA measurements as Merkel et al. (1988), who in their paper make the comment that their approach cannot exclude the existence of small tumour subpopulations expressing P-glycoprotein and that, alternatively, monoclonal antibodies directed against it could be used to detect P-glycoprotein by immunohistochemistry. This is the approach we have chosen. Our results demonstrate that immunocytochemistry may be a useful method for the detection of P-glycoprotein in clinical material. Determination of m-RNA contents (Merkel et al., 1988; Goldstein et al., 1989) may be impractical in the clinical setting for several reasons: it is technically more cumbersome, implies the use of sophisticated laboratory material not present in every hospital and is more time-consuming. Finally, at the present moment, it is still unclear whether only tumour cells express P-glycoprotein under treatment, or if the tumour cells expressing it are the most representative ones for the development of resistance to chemotherapy (Chan et al., 1988). We found, for instance, that ductal cells in a fibroadenomatous area immediately adjacent to the tumour infiltrate in one case, and hyperplastic

**Figure 1** P-glycoprotein reactivity with Mab C219, a1: case no. 21. Positive reaction in tumour cells. Immunoperoxidase × 250. a2: case no. 22. Positive reaction in tumour cells. Immunoperoxidase × 250. a3: negative control of specimen no. 22. Immunoperoxidase × 250. b1: case no. 23. Positive reaction in tumour cells. Immunoperoxidase × 400. b2: case no. 23. Positive reaction in tumour cells. Streptavidin-biotin-phycoerythrin × 400 (method according to Volm *et al.*, 1989*b*). b3: case no. 7. Negative primary tumour of case no. 23 (b1 and b2) before MDR therapy. Immunoperoxidase × 250. c1: daunorubicin-resistant Sa180 nude rat xenografts used as positive control. Immunoperoxidase × 250. c2: case no. 14. Positive reaction in normal ductal cells. Immunoperoxidase × 250. c3: case no. 12. Positive reaction in hyperplastic ductal cells. Immunoperoxidase × 250.

ductal cells in another, expressed P-glycoprotein, whereas the tumour cells themselves did not (Figure 1 c2, c3), a finding that would possibly produce a false-positive result if the same specimens were analysed for m-RNA content. We also had difficulties in attributing any significance to the described pattern of only single tumour cells staining positively for P-glycoprotein in a specimen. At the present state of knowledge we believe that, for practical purposes, these tumours probably should be considered negative. Another finding that caused interpretative trouble was the cytoplasmic staining consistently found in positive cells, although the controls performed in parallel with the reaction ruled out the possibility of unspecific staining. Recently, cytoplasmic P-glycoprotein staining has been reported as a normal finding in multidrug resistant cells at low degrees of resistance (4–6-fold), using a monoclonal antibody (JSB1) that recognises an epitope on the same narrow cytoplasmic domain as the one recognised by C219 (Broxterman *et al.*, 1989). A higher than 10-fold resistance had to be reached in their model tumour cells, before the observed reaction was distinctly membrane bound. Such levels of resistance was probably never reached in humans. Nevertheless, immunofluorescence was able to show in our material that P-glycoprotein is detected mainly on the membrane, even if this is not so apparent in the corresponding immunohistochemical preparation. The results we present also speak in favour of a possible relationship between treatment with multidrug resistance related chemotherapy and the expression of P-glycoprotein in human (mammary) tumours, similar to that found in experimental models (Volm *et al.*, 1989*a*). The one case of this study where P-glycoprotein and clinical resistance appeared in parallel fashion, and the fact that our clearly positive cases were to be found only in the patient group having received MDR therapy in high doses, favour such a hypothesis. The presence of two control groups, one without any treatment and one with non-MDR treatment, where P-glycoprotein was not detected at any significant levels, validates this result.

Its confirmation in larger series and for other tumours would open the possibility for including P-glycoprotein determinations into the planning scheme of tumour therapy. By detecting those tumours which express P-glycoprotein primarily, useless treatment with MDR chemotherapy could be avoided. There is also evidence that the functioning of P-glycoprotein can be effectively modulated by means of certain substances, notably verapamil, trifluoperazine and other membrane-active compounds, although the high doses necessary for obtaining this effect experimentally still preclude their use in patients (Tsuruo et al., 1987).

In conclusion, P-glycoprotein is demonstrable in human mammary carcinomas by means of immunohistochemistry; it was present at clearly detectable levels only after treatment with chemotherapeutic substances related to the multidrug resistance phenomen; finally, its appearance may be related with the development of tumour resistance to MDR therapy. This method may prove to be of use in the future for clinical practice.

J. Schneider is the recipient of a grant from the Spanish Social Security Investigation Fund.

### References

BELL, D.R., GERLACH, J.H., KARTNER, N., BUICK, R.N. & LING, V. (1985). Detection of P-glycoprotein in ovarian cancer: a molecular marker associated with multidrug resistance. *J. Clin. Oncol.*, **3**, 311.

BROXTERMAN, H.J., PINEDO, H.M., KUIPER, C.M. & 7 others (1989). Immunohistochemical detection of P-glycoprotein in human tumour cells with a low degree of drug resistance. *Int. J. Cancer.*, **43**, 340.

CHAN, H.S.L., BRADLEY, G., THORNER, P., HADDAD, G., GALLIC, B.L. & LING, V. (1988). A sensitive method for immunocytochemical detection of P-glycoprotein in multidrug-resistant human ovarian carcinoma cell lines. *Lab. Invest.*, **59**, 870.

GOLDSTEIN, L.J., GALSKI, H., FOJO, A. & 11 others (1989). Expression of a multidrug resistance gene in human cancers. *J. Natl Cancer Inst.*, **81**, 116.

JULIANO, R.L. & LING, V. (1976). A surface glycoprotein modulating drug permeability in Chinese hamster ovary cell mutants. *Biochim. Biophys. Acta*, **55**, 152.

KARTNER, N., RIORDAN, J.R. & LING, V. (1983). Cell surface P-glycoprotein is associated with multidrug resistance in mammalian cell lines. *Science*, **221**, 1285.

KARTNER, N., EVERDEN-PORELLE, D., BRADLEY, G. & LING, V. (1985). Detection of P-glycoprotein in multidrug-resistant cell lines by monoclonal antibodies. *Nature*, **316**, 820.

MA, D.D.F., DAVEY, R.A., HARMAN, D.H. & 5 others (1987). Detection of a multidrug resistant phenotype in acute non-lymphoblastic leukaemia. *Lancet*, **i**, 135.

MERKEL, D.E., FUQUA, S.A.W., HILL, S. & McGUIRE, W.L. (1988). P-glycoprotein gene amplification or overexpression is not detected in clinical breast cancer specimens. In *Prediction of Response to Cancer Therapy*, Hall, C.T. (ed.) p. 61. Alan Liss: New York.

MOSCOW, J. & COWAN, K.H. (1988). Multidrug resistance. *J. Natl Cancer Inst.*, **80**, 14.

SUGAWARA, I., KATAOKA, I., MORISHITA, Y. & 4 others (1988). Tissue distribution of P-glycoprotein encoded by a multidrug resistant gene as revealed by a monoclonal antibody, MRK 16. *Cancer Res.*, **48**, 1926.

THIEBAUT, F., TSURUO, T., HAMADA, H., GOTTESMANN, M.M., PASTAN, I. & WILLINGHAM, M.C. (1987). Cellular localisation of the multidrug resitance gene product P-glycoprotein in normal human tissue. *Proc. Natl Acad. Sci.*, **84**, 7735.

TSURUO, T. (1988). Mechanisms of multidrug resistance and implication for therapy. *Jpn. J. Cancer Res. (Gann)*, **79**, 285.

VOLM, M., EFFERTH, TH., GÜNTHER, A. & LATHAN, B. (1987). Detection of murine S180 cells expressing a multidrug resistance phenotype using different *in vitro* test systems and a monoclonal antibody. *Arzneim.-Forsch./Drug Res.*, **37**, 862.

VOLM, M., BAK, M., EFFERTH, TH, LATHAN, B. & MATTERN, J. (1988*a*). Immunocytochemical detection of a resistance-associated glycoprotein in tissue culture cells, ascites tumors and human tumor xenografts by Mab 265/F4. *Anticancer Res.*, **8**, 531.

VOLM, M., BAK, M., EFFERTH, TH. & MATTERN, J. (1988*b*). Induced multidrug-resistance in murine sarcoma 180 cells grown in vitro and in vivo and associated changes in expression of multidrug-resistance DNA-sequences and membrane glycoproteins. *Anticancer Res.*, **8**, 1169.

VOLM, M., BAK, M., EFFERTH, TH. & MATTERN, J. (1989*a*). Induced multidrug-resistance in murine leukemia L1210 cells and associated changes in a surface-membrane glycoprotein. *J. Cancer Res. Clin. Oncol.*, **115**, 17.

VOLM, M., EFFERTH, TH., BAK, M., HO, A.D. & MATTERN, J. (1989*b*). Detection of the multidrug resistant phenotype in human tumours by monoclonal antibodies and the streptavidin-biotinylated phycoerythrin complex method. *Eur. J. Cancer Clin. Oncol.*, **25**, 743.